# Toward Precision Medicine: Molecular Biomarkers of Response to Tofacitinib in Inflammatory Bowel Disease

**DOI:** 10.3390/genes16080908

**Published:** 2025-07-29

**Authors:** Anja Bizjak, Boris Gole, Gregor Jezernik, Uroš Potočnik, Mario Gorenjak

**Affiliations:** Centre for Human Molecular Genetics and Pharmacogenomics, Faculty of Medicine, University of Maribor, Taborska Ulica 8, 2000 Maribor, Slovenia; anja.bizjak2@um.si (A.B.); boris.gole@um.si (B.G.); gregor.jezernik1@um.si (G.J.); uros.potocnik@um.si (U.P.)

**Keywords:** inflammatory bowel disease, ulcerative colitis, tofacitinib, genomics, transcriptomics, proteomics

## Abstract

Ulcerative colitis (UC), a subtype of inflammatory bowel disease (IBD), is a chronic, relapsing inflammatory condition that significantly impairs the patient’s quality of life. While biologics have transformed disease management, a substantial number of patients remain unresponsive or lose efficacy over time. Tofacitinib (TOFA), an oral Janus kinase (JAK) inhibitor, introduces a novel therapeutic class of small-molecule drugs with a unique oral administration route, offering enhanced patient convenience and broader accessibility compared to parenterally administered biologics. As the first oral treatment approved for moderate to severe UC in years, TOFA acts by modulating the JAK/STAT pathway, influencing critical inflammatory mediators such as IL-6, IL-17, and IFN-γ. However, response rates are variable and appear dose-dependent, with up to 60% of patients showing inadequate therapeutic outcomes. This review represents the first comprehensive synthesis focused specifically on biomarkers of TOFA response in UC. Drawing on multi-omics data—epigenomics, transcriptomics, proteomics, and cellular profiling, we highlight emerging predictors of responsiveness, including CpG methylation signatures (e.g., *LRPAP1* and *FGFR2*), transcriptomic regulators (e.g., *REG3A* and *CLDN3*), immune and epithelial cell shifts, and the cationic transporter MATE1. TOFA demonstrates a dual mechanism by modulating immune responses while supporting epithelial barrier restoration. Despite being promising, TOFA’s dose-dependent efficacy and interpatient variability underscore the critical need for non-invasive, predictive biomarkers to guide personalized treatment. As the first review of its kind, this work establishes a basis for precision medicine approaches to optimize the clinical utility of TOFA in UC management.

## 1. Introduction

Inflammatory bowel disease (IBD) is a chronic, relapsing inflammatory disorder. Even though its pathophysiology remains elusive, it has been established that it arises from the interplay of genetic predisposition, environmental triggers, and disruption of the immune–microbiome axis [1]. The disease is marked by an inappropriate immune response to normal gut stimuli, which leads to mucosal immune dysregulation [2]. Based on the location and the extent of disease’s involvement in the intestinal wall, it is classified into two subtypes: ulcerative colitis (UC) and Crohn’s disease (CD). UC is defined by continuous involvement of the mucosal and submucosal layers of the rectum, extending to varying degrees up the colon. By contrast, CD is a segmental, transmural disease which can occur in any part of the gastrointestinal tract [3,4]. Symptoms of IBD can severely impact a patient’s life and include weight loss, diarrhoea, fatigue, abdominal cramps, and blood in the stool [5,6]. As understanding of IBD pathogenesis grew, elevated levels of the tumour necrosis factor alpha (TNF-α) in serum [7], stool [8], and mucosal biopsy samples [9] gained attention.

### 1.1. From Traditional Immunosuppressive Therapies to More Targeted Treatment Approaches of UC

TNF-α is crucial for maintaining intestinal integrity, but in chronic inflammation, its overproduction significantly contributes to tissue damage and disease progression [10,11]. This dysregulation made TNF-α an effective therapeutic target, leading to the development of anti-TNF biological agents—marking a shift from traditional immunosuppressants like aminosalicylates, immunomodulators, and corticosteroids toward more targeted therapies [12,13,14]. Subsequently, new biologics targeting interleukin-12/23 and α4β7 integrin were introduced [15,16,17,18,19]. While these therapies improved quality of life, they also showed high non-response rates: 11–53% show no initial response, and 23–46% lose efficacy within a year [20,21,22,23,24,25,26]. These limitations underscore the need for more personalized treatment strategies [14].

### 1.2. Treatment Protocols of UC Management

Nowadays, treatment protocols include a wide range of medications, such as anti-inflammatory agents, immune modulators, and, when appropriate, biological drugs that may be added to existing therapies, with surgical intervention remaining an option when needed [27]. Given this complexity, accurate disease assessment and a personalized approach are increasingly important. However, most biomarker panels for biologics rely on invasive mucosal biopsies, limiting patient comfort and clinical utility. Less-invasive methods, like peripheral blood sampling, are needed but remain limited and inconsistent [28].

Biological therapies have improved outcomes for many patients, but their parenteral delivery and immunogenicity risks, along with variable efficacy, present challenges [29,30,31]. As additional treatment options with new mechanisms of action are needed to increase efficacy rates, small-molecule drugs emerged and are paving the way of IBD treatment [32,33]. Unlike biologics, small molecules offer oral delivery and simpler, more cost-effective production [34,35]. A recently developed therapeutic option for the treatment of UC is a small-molecule Janus kinase (JAK) inhibitor, Tofacitinib (TOFA). It has demonstrated significant potential for patients suffering from moderate to severe UC and can be considered a highly effective alternative for those who have failed to respond to biologics or other conventional treatments [32,36,37]. However, research on its mechanisms and predictive biomarkers remains limited. This review aims to synthesize current data and identify key biological pathways and biomarkers linked to TOFA response.

## 2. Tofacitinib

### 2.1. TOFA Mechanism and Development

JAKs are receptor-associated cytosolic tyrosine kinases containing four intracellular enzymes: JAK1, JAK2, JAK3, and TYK2 [38,39]. While most enzymes can be found in almost all tissues, JAK3 is expressed exclusively in the bone marrow and lymphatic system [40,41,42,43]. The JAK family is part of JAK-STAT pathway that mediates external signals and links them to intra-nuclear cascades influencing gene expression [44,45]. Upon receptor binding by a cytokine or hormone, JAKs phosphorylate the receptor, leading to the recruitment and activation of STAT proteins [46]. After the activation, STATs dimerise and translocate to the nucleus, where they regulate the expression of target genes [47].

**Figure 1:**
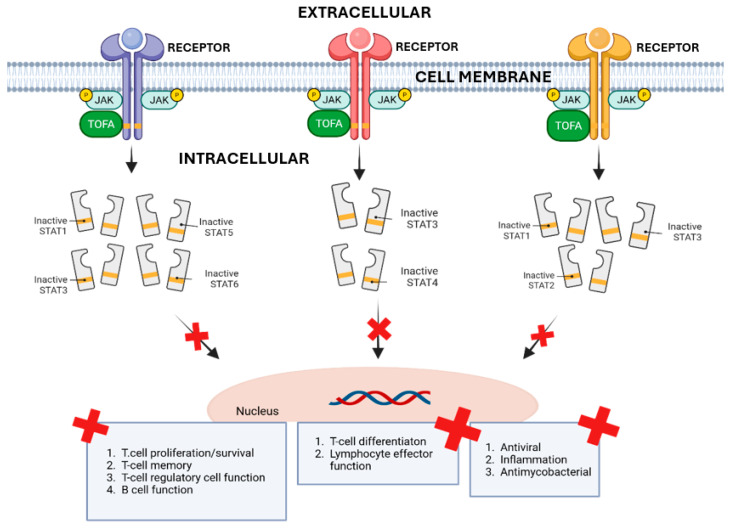
Mechanism of TOFA [48,49,50]. X indicates suppression of this process. (Created with BioRender).

The significance of JAK/STAT pathways in immune response has prompted the exploration of therapeutic strategies involving the pathway’s obstruction to achieve immunosuppression and immunomodulation in the treatment of chronic immune diseases. Through targeted drug design, the JAK1/JAK3 inhibitor was identified. This led to the development of TOFA as a first-in-class JAK inhibitor. Although the goal was 100-fold selectivity for JAK3 over JAK2, the compound ultimately inhibited JAK1 and JAK2 as well, which turned out to be beneficial for broader anti-inflammatory effects, particularly in diseases like rheumatoid arthritis and IBD (Figure 1) [48,49]. The mechanism of TOFA is outlined in Figure 1.

TOFA has demonstrated both safety and efficacy in the treatment of UC, thus earning FDA and EMA approval in the year 2017 [51]. It also represents the first oral therapy for UC to be approved in many years [51,52,53,54,55].

### 2.2. TOFA Limitations

Despite its potential, up to 60% of patients do not respond to TOFA as expected. In elderly individuals, response rates drop to around 50%, likely due to age-related immune remodelling, including B- and T cell immunosenescence and reduced mucosal immunity [56,57]. TOFA is also associated with significant side effects. The most common are herpes zoster and severe infections, but more serious risks include cardiovascular events, malignancies, and thromboembolism [55,58,59,60,61,62]. Regarding pregnancy, TOFA is not recommended due to foeticidal and teratogenic effects observed in animal studies, including fatal malformations and pregnancy loss. Although human data are lacking, its molecular properties suggest the potential for placental transfer [63].

However, in Crohn’s disease, a phase 2b trial, TOFA failed to meet its primary endpoints of clinical remission (defined as Crohn’s Disease Activity Index less than 150), indicating limited effectiveness [64]. These findings emphasize the critical need for more comprehensive safety profiling of TOFA in vulnerable populations, such as the elderly and pregnant women, as well as for the identification of predictive and non-invasive biomarkers to better guide individualized treatment decisions.

## 3. Epigenomics

TOFA targets a key pathway involved in immune regulation and the pathogenesis of IBD. However, patient responses vary, reflecting differences in downstream gene regulation. Genome-wide association studies (GWASs) show that genetic factors explain only about 10% of UC variance [65,66]. This highlights the importance of environmental and epigenetic influences, such as diet, smoking, and antibiotic exposure, which are known to affect the epigenome through mechanisms like DNA methylation [67,68]. Studies have found hypermethylation in genes linked to homeostasis and defence, and hypomethylation in immune-related genes, including chemokines and interleukins [69]. Since TOFA aims to reduce inflammation, these methylation patterns suggest potential as response biomarkers—such as *CXCR1*, *CXCR2*, *CXCL5*, *CXCL6*, *IL17A*, *IL1B*, *REG3A*, *DEFA6*, *SAA1*, *SAA2*, and *LCN2* (Tables S1 and S2) [69]. Additionally, it was shown that hypo-methylation of anti-inflammatory genes is present in severe cases of UC, including *IL10*, *SIGLEC5*, *CD86*, *CLMP*, *NLRP3,* and *NLRC4* [70], indicating a distinctive functional pattern in severe UC, counteracting inflammation. Moreover, this finding may have implications for treatment response assessment in UC.

### Methylation Patterns in Response to TOFA Treatment

As there is an absence of predictive biomarkers for the efficacy of TOFA treatment in UC, a recent longitudinal study investigated peripheral blood genome-wide DNA methylation patterns in response to TOFA treatment and the predictive potential of CpGs [71]. A total of 53 CpG sites in peripheral whole blood DNA have been identified as potential predictors of response to TOFA, with a high degree of accuracy (Table S3) [71]. It is noteworthy that 77% of these CpG markers showed excellent long-term prediction, which means they remained consistent over 20 weeks of TOFA treatment. The study identified hypo- and hypermethylation at specific CpG loci to be correlated with the expression of genes that play a role in various pathways, including JAK/STAT, MAPK, and immune regulation. For example, they observed hypermethylation in a CpG site of the *LRPAP1* gene in responders to TOFA with significant lower gene expression [71]. *LRPAP1* has been shown to code for a chaperone protein, with the capacity to bind calmodulin, which can be phosphorylated by calmodulin-dependent kinase II. This process initiates activation of JAK-STAT signalling pathway [72]. Another gene associated with the JAK-STAT signalling pathway that was found to be hypermethylated in this study was the *FGFR2* gene [71]. *FGFR2* gene is involved in various cellular processes and overexpression of *FGFR2* has been shown to promote STAT1 activation [73].

These findings highlight the potential of epigenomic alterations, particularly DNA methylation changes in immune-regulatory genes, as candidate theragnostic biomarkers for TOFA treatment response in UC. However, to date, no study has specifically investigated genetic polymorphisms associated with TOFA response in UC patients. While genome-wide association studies (GWASs) for TOFA response are lacking, several studies in other biologics—such as anti-TNF—have identified specific SNPs linked to treatment outcomes [74,75,76,77]. These findings support the plausibility of a genomic basis for inter-individual differences in TOFA response.

## 4. Transcriptomic

TOFA acts by modulating cytokine-driven gene transcription via the JAK-STAT pathway; thus, transcriptomic profiling can provide valuable insights into patient responses to treatment and how they can be categorized. Given that RNA transcripts play a central role in immune regulation and inflammation, transcriptomic signatures have already been associated with disease prognosis and activity in UC [78,79,80].

### 4.1. Modulation of Immune-Related Gene Expression by TOFA

TOFA affects both the adaptive and innate immune systems. Flood et al. showed that it transcriptionally downregulates *IL1B* and *IL18* in colonic organoids from IBD and non-IBD patients [81]. Additionally, Ghoreschi et al. showed in a murine model that *IL23R* expression was suppressed in Th17 differentiation models [81,82]. High tissue levels of IL-23, IFN-γ, or IL-6 may limit TOFA’s efficacy, particularly in Th17-skewed inflammation [82]. Th17-dominant profiles are also common in non-responders to anti-TNF-α therapies in Crohn’s disease [28]. This is one reason why TOFA may be more effective in Th1- than in Th17-driven disease, as demonstrated in the studies of Ghoreschi et al. and Ito et al. [82,83]. Ghoreschi’s murine study showed downregulation of key markers for Th1 and Th17 differentiation—*Tbx21*, *Rorc*, *Il23r*, *Il21*, and *Ahr*—following TOFA treatment [82]. These molecules define the identity and functional programming of Th1 and Th17 cells as described in the review by Muranski and Restifo [84].

### 4.2. Biomarkers of Tofacitinib Response: Responders vs. Non-Responders

The suppression of *Tbx21* and STAT1 signalling in Ghoreschi et al.’s study suggests a strong effect of TOFA on Th1 responses, aligning with findings by Ito et al. Their analysis of biopsy samples from responsive and non-responsive patients showed significant downregulation of *Tbx21*, a key Th1 transcription factor, following TOFA treatment. This supports the idea that TOFA is more effective in modulating Th1 responses, while persistent Th17 activity may contribute to non-response [83].

In addition, several studies have examined transcriptomic markers of TOFA to improve the prediction of therapeutic outcomes in UC [61,80,83]. Profiling of fibroblasts and macrophages from responders revealed a distinct signature, including reduced expression of *S100A9*, a pro-inflammatory calprotectin subunit. Responders also showed downregulation of JAK1, STAT1, and interferon-response genes like *GBP1*, *IFITM3*, and *ISG15*, indicating suppressed inflammation. At the same time, elevated *AHR*, *IGF1*, *MAF*, and *IL10RA* levels pointed to tissue repair and immune regulation, suggesting potential biomarkers of favourable TOFA response [61,85,86,87,88]. Interestingly, the *Ahr* gene was found to be downregulated in murine models, whereas its expression was upregulated in human samples, indicating a potential species-specific regulation or context-dependent role in inflammatory pathways.

### 4.3. Dual Role of TOFA at the Transcriptomic Layer

**Figure 2:**
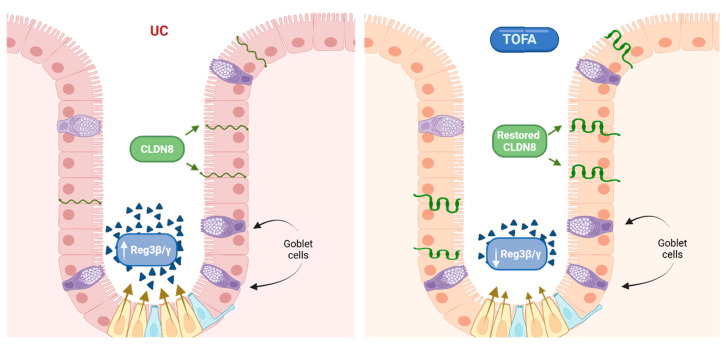
Epithelial defence potential of TOFA [89,95]. (Created with BioRender).

The potential of RNA molecules as biomarkers for therapeutic response is also suggested by other transcriptomic studies [89,90]. In the context of UC, the restoration of mucosal integrity represents a central therapeutic objective [91,92]. TOFA’s ability to promote both immune modulation and epithelial repair underscores its promising dual role. Gonneaud’s study showed that TOFA modulates genes involved in epithelial homeostasis. *Alpi*, a relevant gene, was upregulated in murine models [89]. It has been previously demonstrated that the reduction in ALPI expression in patients suffering from IBD is one of the key mechanisms by which the disease exerts its psychological impact [93]. The study also found increased expression of antimicrobial peptides *Reg3g* and *Reg3b*, produced by Paneth cells, suggesting enhanced mucosal defence [89,94]. Similarly, Komeda et al. reported restored epithelial integrity in UC patients treated with TOFA [95]. However, while Gonneaud et al. observed upregulation of *Reg3g* and *Reg3b* in mice, Komeda found TOFA-induced downregulation of their human homologs—*REG1A*, *REG1B*, and *REG3A*. Since REG upregulation is associated with persistent inflammation in UC, their downregulation may reflect TOFA’s anti-inflammatory efficacy and potential to support mucosal healing [95,96]. Komeda also reported *CLDN8* as one of the top upregulated transcripts. This gene encodes a tight junction protein expressed in intestinal epithelial cells, suggesting enhanced epithelial barrier integrity following TOFA treatment [95,97,98]. The proposed mechanism by which TOFA modulates epithelial defence in UC patients is illustrated in Figure 2.

An additional, though less-studied, group of epithelial molecules relevant to UC is the CEACAM adhesion proteins. These act as receptors for bacterial ligands, triggering immune responses and contributing to intestinal barrier disruption [99,100]. Furthermore, the dysregulation has been observed in IBD, making them a possible target for intestinal barrier renewal [101,102,103]. A recent study examined *CEACAM* expression in IBD patients and found elevated levels of *CEACAM3*, *CEACAM5*, and *CEACAM6* in UC colon samples compared to controls. These levels were significantly reduced after TOFA treatment, although the effect appeared time-dependent and may not persist long-term [103].

### 4.4. Potential Predictive Transcriptomic Biomarkers of Response to TOFA Treatment

To evaluate drug response and optimize treatment, Jang et al. identified *SLC47A1* as a potential predictor of TOFA responsiveness using patient-derived intestinal organoids. This gene encodes MATE1, a cationic transporter linked to improved clinical outcomes in UC. Lower MATE1 expression was observed in non-responders, indicating reduced TOFA sensitivity [90]. The *MATE1* gene is primarily expressed in the kidney and liver, and functions as the principal mediator of the efflux of organic cations [104]. The potential of *MATE1* as a prognostic biomarker of drug response has also been highlighted in recent studies across various diseases [105,106]. Additionally, methylation-related expression changes have been identified in *FGFR2*, *LRPAP1*, and *OR2L13* [71]. These genes are involved in epithelial homeostasis and immune modulation, suggesting functional impacts from DNA-level changes [107,108]. *OR2L13*, an olfactory receptor gene, is expressed in the gastrointestinal tract, where it may help immune or epithelial cells respond to microbial or metabolic cues, influencing inflammation [109,110]. Table 1 summarizes the correlation between transcriptomic markers and TOFA treatment response in UC.

### 4.5. The Role of miRNAs as Predictors of TOFA Treatment Response

As important gene expression regulators, micro RNAs (miRNAs) are involved in regulating the expression of most human genes and play an important role in the pathogenesis of many autoimmune diseases including UC [111,112,113]. There have been several studies reporting dysregulation of miRNAs in IBD, thus proposing new potential targets [111,112,114,115,116,117]. We found no studies reporting miRNA biomarkers specifically linked to TOFA treatment in IBD. However, recent research has explored miRNAs as predictors of TOFA response in rheumatoid arthritis (RA). Since RA and IBD share key immunopathological features—such as chronic inflammation, immune dysregulation, and overlapping cytokine and genetic profiles—it is plausible that miRNAs relevant to TOFA response in RA may also apply to IBD. This cross-disease evidence highlights the need to investigate miRNA-based biomarkers in IBD, which could reveal shared or distinct regulatory mechanisms and aid in identifying predictors of treatment response, supporting personalized therapy.

## 5. Proteomics

In IBD, several conventional protein-based markers—such as C-reactive protein (CRP), albumin, and faecal calprotectin, are routinely used to assess disease severity and inflammation. These biomarkers, while useful, provide only a partial view of the complex molecular processes involved.

Proteomic profiling offers critical insights into biological processes, as most gene functions are executed through proteins [118]. In TOFA treatment, proteomic data are particularly valuable for understanding its mechanisms and identifying response biomarkers. Studies emphasize the benefit of combining transcriptomic and proteomic profiling to assess TOFA’s effects in UC [61,82,83,90]. Flood et al. and Ghoreschi et al. showed that TOFA’s impact extends beyond transcriptomics to include functional protein-level changes, validating transcriptomic markers biologically. TOFA has been shown to suppress IL-1β and IL-18 at both gene expression and cytokine levels, along with downstream cytokines related to Th17 and Th1 differentiation [81,82]. It also reduces secretion of IFN-γ and IL-13 and regulates transcription of key Th1/Th2 markers such as T-bet and GATA3. These findings confirm TOFA’s broad immunomodulatory effects across T cell subsets, supporting its therapeutic utility in UC [82].

### 5.1. The Complexity of the TOFA Mechanism in Inflammation

UC was once considered a primarily Th2-driven disease, but recent findings reveal a more complex T cell landscape. TOFA has shown efficacy in Th1-driven inflammation but reduced effects in Th17-dominant cases [119,120,121]. Studies by Melón-Ardanaz et al. and Ghoreschi et al. indicate that Th17 cells are highly enriched in inflamed UC tissue, and TOFA may, in some non-responders, inadvertently contribute to inflammation via cytokines like IL-17A [83] and IL-22 [61,82,122]. Notably, Th17 cells exhibit functional plasticity, with some shifting toward a Th1-like phenotype and co-producing IFN-γ [82], IL-2 [82], and TNF-α [123,124], which can contribute to the occurrence and severity of tissue damage. [125] TOFA impacts these cytokines, suggesting partial efficacy even in Th17-skewed environments.

However, TOFA’s cytokine effects are not consistently suppressive and appear dose-dependent [123,124]. Cordes et al. showed that in GM-CSF–differentiated monocytes, high-dose TOFA (1 µM) significantly suppressed TNF-α, IL-6, and IL-10, while an intermediate dose (0.1 µM) had no notable effect on IL-10, though TNF-α suppression persisted [124]. Studies have shown that monocytes are an important mediator of chronic inflammatory diseases due to their ability to infiltrate inflamed tissue and differentiate into cytokine-producing macrophages [126,127]. Lethen et al. examined the effects of TOFA on circulating monocyte-derived macrophages, including M1- and M2-like phenotypes from both healthy donors and IBD patients [123]. M1 macrophages promote inflammation while M2 types support wound healing [128]. The study found a dose-dependent response: low doses (1–5 µM) modestly increased TNF-α; high doses (10 µM) reduced TNF-α but elevated IL-1β and IL-23. All doses enhanced IL-10 in M1 macrophages. However, TOFA impaired M2-like polarization and reduced protective IL-10 secretion [123]. This contrasts with Cordes et al., where high doses either inhibited or had no effect on IL-10 [124]. These discrepancies likely reflect variations in polarization state when TOFA was administered and the dosages used.

### 5.2. Dual Role of TOFA at the Proteomic Layer

Proteomic profiles also support the role of TOFA in promoting intestinal epithelial barrier regeneration. As noted earlier, genes related to barrier function showed partial restoration following TOFA treatment. This transcriptomic improvement was validated by Gonneaud et al., who reported restored expression of the CLDN3 protein in murine models [89]. CLDN3 is one of the tight junction proteins that regulates epithelial homeostasis [98]. In a recent study, the combination of TOFA and corticosteroid budesonide led to increased protein levels of LGR5 and, to a lesser extent, EPHB2 [129]. LGR5, expressed on intestinal stem cells, is essential for epithelial renewal, while EPHB2 influences immune function and cell migration. Disruptions in T cell differentiation are linked to intestinal inflammation and poor mucosal healing [130,131,132,133]. Inflammatory cytokines contribute to this impairment by creating a microenvironment hostile to epithelial regeneration. In addition to transcriptomic and epigenomic evidence, proteomic data also confirm TOFA’s suppression of key cytokines such as IL-6, IL-17, and IFN-γ [134]. The link between these cytokines and both the risk and pathogenesis of IBD is well established [135,136,137,138].

## 6. Cells as Biomarkers

### 6.1. Different Cell Biomarkers of Response to TOFA in Responders vs. Non-Responders

To identify cellular subsets associated with TOFA response and non-response, Melón-Ardanaz et al. conducted an analysis of biopsy samples from subjects prior to and following therapy [139]. Single-cell RNA-seq (scRNA-seq) was used to assess immune cell dynamics in UC patients treated with TOFA. Responders showed a marked reduction in pro-inflammatory cell types, including inflammatory macrophages, plasma cells, neutrophils, and fibroblasts, alongside increases in epithelial and stromal cells linked to tissue repair. In contrast, non-responders exhibited an accumulation of inflammatory myeloid cells and macrophages with a strong pro-inflammatory profile. Not only did TOFA fail to resolve inflammation in these patients, but it may have amplified pro-inflammatory signalling in macrophages [139]. This non-response mechanism was further explored in a follow-up study [61]. Using the Pathway RespOnsive GENes method on scRNA-seq data, researchers found elevated pro-inflammatory macrophages and fibroblasts in non-responders, along with increased NF-kB pathway activation—a key driver of inflammation, immune response, and cell survival. TOFA’s effect on epithelial integrity was also emphasized in related findings [61,140,141]. The heightened NF-kB signalling in non-responders supports a persistent inflammatory process, aligning with prior results from 2023. In responders, significant reductions in plasma cells, lymphocytes, granulocytes, and stromal cells were observed in tissue samples, though no change in circulating leukocytes was noted. Additional analysis confirmed a decrease in myeloid, B-, and plasma cells, coupled with increases in epithelial and stromal cell populations. Myeloid subtypes such as macrophages and fibroblasts showed anti-inflammatory remodelling. A 2025 study by Melón-Ardanaz et al. reinforced TOFA’s therapeutic role, showing it downregulates inflammatory genes and restores a healthy intestinal macrophage and fibroblast signature [61].

### 6.2. Cells as Predictors of Response to TOFA Treatment

Ito et al. identified IL-17–positive mononuclear cells as potential predictors of TOFA unresponsiveness, supported by both proteomic and transcriptomic analyses [83]. In epithelial lineages, Gonneaud et al. demonstrated in mice that TOFA treatment increased Paneth cell numbers in jejunal crypts, suggesting a link between TOFA response and specific cellular markers [89]. Interestingly, no increase in goblet cells was observed, despite both goblet and Paneth cells originating from intestinal stem cells and contributing to mucosal defence [142]. This may indicate that TOFA influences intestinal stem cell differentiation. Table 2 summarizes all cell-type markers associated with TOFA treatment discussed in this review.

Another study showed that TOFA regulates both innate and adaptive immune cells. In murine models, Th1, Th2, and Th17 cells were identified as biomarkers of TOFA’s immunomodulatory effects [82]. TOFA disrupted Th1 and Th2 differentiation and reduced inflammatory Th17 cell production [82]. More recent studies also demonstrated TOFA’s influence on innate immune cell polarization and T cell interactions [123,124]. A study by Cordes et al. reported that TOFA can reprogram monocytes towards a regulatory phenotype [124]. Lethen et al. focused on macrophages and showed that TOFA modulates polarization by suppressing pro-inflammatory M1 macrophages (CD80, CD86, and CD40 markers) and altering M2 features, notably reducing CD206 expression. CD163 expression increased slightly at low TOFA doses but decreased at higher concentrations [123].

## 7. Correlation of Epigenetically Altered Genes in UC with Biomarkers of TOFA Response

Our literature review identified several TOFA response biomarkers in UC that overlap with genes annotated to differentially methylated regions in the study by Taman et al., as shown in Table 3 [70,80]. Komeda et al. reported multiple TOFA response markers aligning with Taman et al.’s findings. Three genes—*SLC6A19*, *CLDN8*, and *HMGCS1*—were downregulated in untreated UC patients but ranked among the top upregulated genes post-TOFA treatment [80,95]. These genes are crucial for maintaining epithelial barrier integrity [143,144,145]. Conversely, three genes identified by Taman et al. as significantly downregulated due to hypermethylation were also downregulated in TOFA responders, possibly reflecting a regulated reduction in epithelial turnover during inflammation resolution. Moreover, five genes upregulated by hypomethylation in UC were found to be downregulated by TOFA in Komeda’s study [80,95]. Melon et al. identified *IL1B* and *S100A9* as downregulated transcriptomic markers in TOFA responders, both of which are also among the most hypomethylated genes in UC [61,80]. These genes have known pro-inflammatory roles [146,147], while *CHI3L1* is linked to epithelial stress and barrier dysfunction [148]. Additionally, several proteomic biomarkers associated with TOFA treatment correlate with Taman et al.’s methylation findings (Table 3). For instance, Lethen et al. showed increased IL-1β release [123], while other studies reported its suppression by TOFA [81]. CXCL5 and CXCL6 have also been identified as biomarkers of TOFA response, though their regulation remains inconsistent, highlighting the complexity of the underlying biology [129].

### Evidence Supporting TOFA’s Dual Mechanism of Action

The study by Sridhar et al. demonstrated a reduction in the expression of CXCL5, but an increase in the expression of CXCL6 cytokine [129]. While both are neutrophil attractants, CXCL5 is JAK/STAT pathway-dependent [149,150] and CXCL6 is regulated by multiple pathways [151,152]. Interestingly, despite their roles in inflammation, upregulation of both cytokines has also been associated with tissue repair [153], suggesting that their expression in the context of TOFA treatment may reflect a complex balance between dampening inflammation and promoting mucosal healing. Table 3 illustrates the correlation between epigenetically altered genes in UC and biomarkers that respond to TOFA.

## 8. Future Challenges

Therapeutic advances have been made in the treatment of ulcerative colitis (UC), although the condition continues to have a significant impact on patients’ quality of life [154,155]. Despite the considerable impact of biological therapies on the management of inflammatory bowel diseases, a proportion of patients either fail to respond initially or lose response over time [156,157,158]. To overcome these limitations, drug development has shifted to small-molecule drugs. TOFA was the first small-molecule drug of the next generation to be approved for use in UC treatment [52]. However, there are still several unclear areas that require further investigation. Small-molecule drug TOFA is regarded as one of preferred treatment options in managing UC. Nevertheless, it does not eliminate the challenges associated with UC.

### 8.1. Current Limitations in TOFA Treatment of UC

Despite the convenience of oral delivery, TOFA faces challenges such as poor water solubility, limited bioavailability, and dissolution-limited absorption, which can hinder its effectiveness [159]. These factors may lead to inconsistent drug levels at inflamed sites, reducing efficacy, and potentially necessitating higher or more frequent dosing [160]. Its dose-dependent action raises concerns, as higher doses can increase adverse effects [57,123,124,161].

An additional factor that must be carefully considered is patient-specific variability, such as microbiome composition, age, and disease severity, as these can significantly influence the effectiveness, safety, and pharmacokinetics of TOFA [162,163]. Although pharmacological properties like solubility and metabolic profile contribute to a drug’s effectiveness, patient-specific biological factors frequently serve as the primary determinants of therapeutic response [159]. Currently there has been no universal validation of a single definitive biomarker as the most reliable indicator of efficacy to TOFA. Nonetheless, studies have suggested some promising biomarkers indicating response to TOFA treatment.

TOFA is primarily metabolized by CYP3A4, but liver enzyme activity varies between individuals [164,165]. Gastrointestinal conditions like dysbiosis, mucosal damage, and body mass can also affect absorption [163,166]. Obesity may reduce bioavailability due to increased inflammatory burden [166,167]. Furthermore, the significance of the inflammatory burden in the treatment of TOFA is corroborated by studies conducted by Melón-Ardanaz et al. and Cordes et al., indicating that the baseline immune cell populations of patients, particularly inflammatory macrophages, T cells, and fibroblasts, serve as significant predictors of the response to TOFA [61,124]. In cases of non-response, a tendency towards immune cell profiles that are resistant to JAK inhibition is frequently observed. Such profiles are pro-inflammatory in nature [61,124]. Moreover, the persistence of Th17/IL-17A+ T cells and NF-κB activation in non-responders indicates that JAK inhibition alone may prove insufficient to suppress Th17-dominant inflammation, suggesting the potential presence of a complex cellular resistance mechanism underlying non-response [83]. Interestingly, Th17 cells are also well-documented players of non-response to biologics like adalimumab [168]. As Kobeissy et al. demonstrated, the suppression of the NF-κB pathway by miR-27a-5 could be a promising adjunctive therapy for non-response targeting the NF-κB pathway [169].

### 8.2. Unmet Needs and Challenges in Predictive Biomarkes to TOFA Treatment

Given these challenges, integrating predictive biomarkers into clinical decisions offers strong potential to improve outcomes. Joustra et al. proposed using peripheral blood DNA methylation profiles as a minimally invasive tool for patient stratification [71]. While they identified 53 CpG loci associated with TOFA response, most were intronic and lacked corresponding gene expression changes, limiting their interpretability. Only *LRPAP1* and *OR2L13*—both involved in inflammatory response—showed differential expression. The study also relied on the MAYO endoscopic score, which has limitations, including lack of validation and variability in friability assessment [71,170]. Additionally, endoscopy visualizes only the superficial mucosa, limiting its ability to assess deeper disease activity [171]. Despite these limitations, the study provides valuable insight into TOFA response. Further research with larger, independent cohorts is needed to clarify the functional significance of these findings.

### 8.3. Lack of miRNA Studies in Response to TOFA Treatment

We believe that a further shortcoming in the investigation of the TOFA effect in UC is the absence of research on non-coding RNA (ncRNA). NcRNAs are a functional RNAs that are not translated into a protein. It has been established that these elements exert a considerable influence on the process of gene regulation as they are involved in the process of regulating gene expression at both, the transcriptional and post-transcriptional levels. In 2011, Salmena et al. proposed an alternative mechanism of gene regulation, suggesting that numerous natural RNA molecules can act as “sponges” for miRNAs by competing for binding, a phenomenon referred to as “sponge RNA” [172]. In the recent years, they also demonstrated this mechanism in IBD pathology but have not yet explored its application in IBD therapy [173,174]. Nevertheless, the majority of ncRNAs being studied are miRNAs, although other ncRNAs, such as long ncRNAs (lncRNAs) and circular RNAs (circRNAs), are also starting to show important contributions to human diseases [175]. When it comes to identifying potential ncRNA biomarkers for TOFA, we found a few studies on miRNAs regarding TOFA treatment in RA. The shared inflammatory pathways between RA and IBD suggest that these miRNAs represent promising candidates for further exploration as biomarkers of response to TOFA in UC [176,177,178].

### 8.4. TOFA Beyond Immunosuppression

Finally, it is crucial to emphasize that, when considered collectively, studies are indicative of the dual effect of TOFA. Although it was originally developed for the suppression of JAK1/JAK3 signalling, recent studies have demonstrated a more extensive impact, such as epithelial restoration. This has been shown using a combination of transcriptomic, proteomic, and cell markers [89,95,169]. Gonneaud’s study identified two key findings. Firstly, the restoration of antimicrobial peptide REG3G and transmembrane tight junction protein CLDN3 was identified following treatment with TOFA. Secondly, the study revealed an increase in Paneth cells, a specific type of intestinal epithelial cell [89]. Reg3 proteins are predominantly expressed in Paneth cells; therefore, this combination of protein and cellular markers could indicate a more selective epithelial remodelling effect of TOFA [94]. The extension of scRNA-seq to include epithelial subtypes would be of significant benefit, as demonstrated in the study by Melón-Ardanaz et al. [139]. This approach has the potential to generate more profound insights into the response by TOFA treatment intestinal epithelial remodelling.

A translational gap remains between in vitro findings and clinical application. Studies by Sridhar et al. and Lethen et al. and Saiz-Gonzalo et al. have shown that organoid and immune cell models provide mechanistic insights [103,123,129]. However, organoids remain reductionist, lacking immune–epithelial interactions. Genomic biomarkers predicting TOFA response still show inconsistent significance and reproducibility. Greater alignment between genomic, transcriptomic, and proteomic biomarkers is needed to fully understand drug response and improve patient stratification [179,180]. This review identified several transcriptomic and proteomic biomarkers of TOFA response that overlap with differentially methylated regions previously reported in treatment-naïve UC patients. This overlap suggests a biological link between UC pathogenesis and TOFA response, underscoring the value of integrating multi-omics data across disease stages to identify reliable predictive markers.

## 9. Conclusions

While many CpG loci, transcriptomic signatures, and proteomic biomarkers have been linked to TOFA response, their greatest utility lies in integrated multi-omics approaches. Peripheral blood DNA methylation profiling—especially at stable loci—offers a minimally invasive and scalable diagnostic option. When combined with transcriptomic data from biopsies or blood and cytokine-focused proteomic profiling, these layers can improve biomarker panel accuracy. This integrated strategy enhances responder stratification by capturing regulatory dynamics across biological systems.

To implement these findings clinically, prospective validation, standardized sample handling, and user-friendly diagnostic platforms are essential. Ultimately, such tools could help stratify treatment-naïve patients, reduce exposure to ineffective therapies, and advance precision medicine in UC.

## Figures and Tables

**Table 1:** Transcriptomic markers associated with treatment response to tofacitinib in ulcerative colitis.

Gene Symbol	Full Gene Name	Tissue	TOFA Regulation	Reference
*Il1b* (mouse)	Interleukin 1 Beta	naive murine CD4(+) T cells	Down	[82]
*Il18* (mouse)	Interleukin 18	naive murine CD4(+) T cells	Down	[82]
*Tbx21* (mouse)	T-Box Transcription Factor 21	naive murine CD4(+) T cells	Down	[82]
*Rorc* (mouse)	RAR-Related Orphan Receptor gamma	naive murine CD4(+) T cells	Down	[82]
*Il23r* (mouse)	Interleukin 23 Receptor	naive murine CD4(+) T cells	Down	[82]
*Il21* (mouse)	Interleukin 21	naive murine CD4(+) T cells	Down	[82]
*Ahr* (mouse)	Aryl Hydrocarbon Receptor	naive murine CD4(+) T cells	Down	[82]
*TBX21*	T-Box Transcription Factor 21	peripheral T cells	Down	[83]
*ISG15*	ISG15 Ubiquitin-Like Modifier	intestinal macrophages	Down	[61]
*GBP1*	Guanylate Binding Protein 1	intestinal macrophages	Down	[61]
*S100A9*	S100 Calcium-Binding Protein A9	intestinal macrophages	Down	[61]
*IFITM3*	Interferon-Induced Transmembrane Protein 3	intestinal macrophages	Down	[61]
*AHR*	Aryl Hydrocarbon Receptor	intestinal macrophages	Up	[61]
*IGF1*	Insulin-Like Growth Factor 1	intestinal macrophages	Up	[61]
*MAF*	MAF BZIP Transcription Factor	intestinal macrophages	Up	[61]
*IL10RA*	Interleukin 10 Receptor Subunit Alpha	intestinal macrophages	Up	[61]
*Alpi* (mouse)	Alkaline Phosphatase, Intestinal	enteroid cultures from murine crypts	Up	[89]
*Cldn3* (mouse)	Claudin 3	enteroid cultures from murine crypts	Up	[89]
*Reg3g* (mouse)	Regenerating Islet-Derived 3 Gamma	enteroid cultures from murine crypts	Up	[89]
*Reg3b* (mouse)	Regenerating Islet-Derived 3 Beta	enteroid cultures from murine crypts	Up	[89]
*CLDN8*	Claudin 8	rectal mucosa	Up	[95]
*REG1A*	Regenerating Family Member 1 Alpha	rectal mucosa	Down	[95]
*REG1B*	Regenerating Family Member 1 Beta	rectal mucosa	Down	[95]
*REG3A*	Regenerating Family Member 3 Alpha	rectal mucosa	Down	[95]
*CEACAM3*	CEA Cell Adhesion Molecule 3	colonic biopsies	Down	[103]
*CEACAM5*	CEA Cell Adhesion Molecule 5	colonic biopsies	Down	[103]
*CEACAM6*	CEA Cell Adhesion Molecule 6	colonic biopsies	Down	[103]
*FGFR2*	Fibroblast Growth Factor Receptor 2	peripheral blood	Down	[71]
*LRPAP1*	LDL Receptor-Related Protein Associated Protein 1	peripheral blood	Down	[71]
*OR2L13*	Olfactory Receptor Family 2 Subfamily L Member 13	peripheral blood	Up	[71]
*SLC47A1*	Solute Carrier Family 47 member 1	colonic biopsies	Up	[90]

**Table 2:** Cell-specific markers associated with tofacitinib treatment response in ulcerative colitis.

Cell Type	Regulation	R/NR	References
Inflammatory macrophages	Down	R	[139]
Plasma cells	Down	R	[61,139]
Neutrophils	Down	R	[139]
Inflammatory fibroblasts	Down	R	[139]
Epithelial cells	Up	R	[61,139]
Stromal cells	Up	R	[61,139]
Myeloid cells	Up	NR	[61,139]
Inflammatory macrophages	Up	NR	[61,139]
Inflammatory fibroblasts	Up	NR	[61]
B-lymphocytes	Down	R	[61]
Granulocytes	Down	R	[61]

R: responders; NR: non-responders.

**Table 3:** Correlation between epigenetically altered genes in ulcerative colitis and tofacitinib treatment response biomarkers.

Gene Symbol	UC Naïve Regulation	TOFA Regulation	Type of TOFA Biomarker	References
*CLDN8*	Down	Up	T	[80,95]
*GUCA2A*	Down	Down	T	[80,95]
*HAVCR1*	Down	Down	T	[80,95]
*HMGCS2*	Down	Up	T	[80,95]
*PCK1*	Down	Down	T	[80,95]
*SLC6A19*	Down	Up	T	[80,95]
*CHI3L2*	Up	Down	T	[61,80]
*CXCL5*	Up	Down	T, P	[80,95,129]
CXCL6	Up	Up	P	[80,129]
IL17A	Up	Down	P	[80,134]
*IL1B*	Up	Up/Down	T, P	[61,80,81,123]
*REG1B*	Up	Down	T	[80,95]
*REG3A*	Up	Down	T	[80,95]
*S100A9*	Up	Down	T	[61,80]
*SAA2*	Up	Down	T	[80,95]
*SLC26A4*	Up	Down	T	[80,95]

T: transcriptomic marker; P: proteomic marker.

## Data Availability

No new data were created or analyzed in this study.

## Supplementary Materials

The following supporting information can be downloaded at: https://www.mdpi.com/article/10.3390/genes16080908/s1, Table S1: TOP hypermethylated genes in UC as reported by Taman et al.; Table S2: TOP hypomethylated genes in UC as reported by Taman et al.; Table S3: TOP CpGs predictors of response to TOFA reported by Joustra et al.

## Abbreviations

The following abbreviations are used in this manuscript:

CD	Crohn’s Disease
circRNA	Circular RNA
CpG	Cytosine-phosphate-Guanine site
EMA	European Medicines Agency
FDA	Food and Drug Administration
GWASs	Genome-Wide Association Studies
IBD	Inflammatory Bowel Disease
lncRNA	Long Non-coding RNA
miRNA	MicroRNA
ncRNA	Non-coding RNA
NR	Non-Responder(s)
R	Responder(s)
RA	Rheumatoid Arthritis
scRNA-seq	Single-cell RNA sequencing
SNP	Single Nucleotide Polymorphism
Th1	T-helper 1 cells
Th17	T-helper 17 cells
TNF-α	Tumour Necrosis Factor Alpha
TOFA	Tofacitinib
UC	Ulcerative Colitis
