# Supplementary material for: Toward Precision Medicine: Molecular Biomarkers of Response to Tofacitinib in Inflammatory Bowel Disease
Source: Genes (Basel). 2025 Jul 29;16(8):908. doi: 10.3390/genes16080908 (PMC12385345; doi:10.3390/genes16080908)
Supplement: Supplementary file 1 [file genes-16-00908-s001.zip › Table_S1.pdf]

**Table S1**: TOP hypermethylated genes in UC as reported by Taman et al. [69,70]

| Gene symbol | Gene name | % methyl | #c | Ref |
|---|---|---|---|---|
| *ADIRF* | Adipogenesis Regulatory Factor | -5,72 | 36 | [69] |
| *AGMO* | Alkylglycerol Monooxygenase | -10,86 | 7 | [69] |
| *ALPI* | Alkaline Phosphatase, Intestinal | -3,05 | 34 | [69] |
| *ANKRD62* | Ankyrin Repeat Domain 62 | -0,19 | 91 | [69] |
| *BCHE* | Butyrylcholinesterase | -4,17 | 10 | [69] |
| *BRINP3* | BMP/Retinoic Acid Inducible Neural Specific 3 | -0,22 | 29 | [69] |
| *CES2* | Carboxylesterase 2 | -4,39 | 293 | [70] |
| *CLDN8* | Claudin 8 | -3,56 | 6 | [69] |
| *CYP3A4* | Cytochrome P450 Family 3 Subfamily A Member 4 | -15,45 | 2 | [69] |
| *SNORC* | Secondary Ossification Center Associated Regulator of Chondrocyte Maturation | -21,84 | 54 | [70] |
| *AKAP19* | A-kinase Anchoring Protein 19 | -5,89 | 13 | [70] |
| *DEFB1* | Defensin Beta 1 | -4,83 | 16 | [69] |
| *DRAIC* | Downregulated RNA in Cancer, Inhibitor of Cell Invasion and Migration | -20,47 | 45 | [70] |
| *ENTPD5* | Ectonucleoside Triphosphate Diphosphohydrolase 5 (inactive) | -22,12 | 28 | [70] |
| *FABP1* | Fatty Acid Binding Protein 1 | -16,56 | 18 | [69] |
| *FAM151A* | Family With Sequence Similarity 151 Member A | -3,59 | 6 | [69] |
| *FRMD1* | FERM Domain Containing 1 | -2,08 | 109 | [69] |
| *GBA3* | Beta-Glucosidase 3 | -14,5 | 7 | [69] |
| *GUCA2A* | Guanylate Cyclase Activator 2A | -12,84 | 18 | [69] |
| *GUCA2B* | Guanylate Cyclase Activator 2B | -9,12 | 41 | [69] |
| *HAVCR1* | Hepatitis A Virus Cellular Receptor 1 | -12,55 | 26 | [69] |
| *HMGCS2* | 3-Hydroxy-3-Methylglutaryl-CoA Synthase 2 | -13,13 | 18 | [69] |
| *HSD17B2* | Hydroxysteroid 17-Beta Dehydrogenase 2 | -2,69 | 6 | [69] |
| *MAGIX* | MAGI Family Member, X-Linked | -19,44 | 107 | [70] |
| *MEP1A* | Meprin A Subunit Alpha | -11,49 | 18 | [69] |
| *MMP28* | Matrix Metallopeptidase 28 | -3,99 | 112 | [70] |
| *NGEF* | Neuronal Guanine Nucleotide Exchange Factor | -15,9 | 42 | [70] |
| *OTC* | Ornithine Carbamoyltransferase | -6,32 | 15 | [69] |
| *PCK1* | Phosphoenolpyruvate Carboxykinase 1 | -6,73 | 8 | [69] |
| *PNLIPRP2* | Pancreatic Lipase Related Protein 2 | -15,2 | 26 | [69] |
| *P3H2* | Prolyl 3-hydroxylase 2 | -2,41 | 21 | [70] |
| *PFKFB2* | 6-Phosphofructo-2-Kinase/Fructose-2,6-Biphosphatase 2 | -4,59 | 163 | [70] |
| *PRAP1* | Proline Rich Acidic Protein 1 | -3,12 | 260 | [69] |
| *PPARGC1A* | PPARG Coactivator 1 Alpha | -6,09 | 42 | [70] |
| *PRKG2* | Protein Kinase cGMP-Dependent 2 | -21,46 | 100 | [70] |
| *NECTIN3* | Nectin Cell Adhesion Molecule 3 | -22,71 | 30 | [70] |
| *SLC17A8* | Solute Carrier Family 17 Member 8 | -2,89 | 5 | [69] |
| *SLC67A1-AS* | SLC67A1 Antisense RNA | -15,49 | 115 | [70] |
| *SLC22A4* | Solute Carrier Family 22 Member 4 | -0,39 | 172 | [69] |
| *SLC25A34* | Solute Carrier Family 25 Member 34 | -2,53 | 10 | [69] |
| *SLC30A10* | Solute Carrier Family 30 Member 10 | -7,67 | 14 | [69] |
| *SLC3A1* | Solute Carrier Family 3 Member 1 | -11,12 | 40 | [69] |
| *SLC51B* | Solute Carrier Family 51 Subunit Beta | -19,92 | 33 | [70] |
| *SLC6A19* | Solute Carrier Family 6 Member 19 | -12,17 | 13 | [69] |
| *SULT1A2* | Sulfotransferase Family 1A Member 2 | -15,79 | 7 | [69] |

| | | | | |
|---|---|---|---|---|
| *TMEM72* | Transmembrane Protein 72 | -14,90 | 58 | [70] |
| *TRPM4* | Transient Receptor Potential Cation Channel Subfamily M Member 4 | -30,69 | 109 | [70] |
| *TINCR* | TINCR Urothelial Differentiation Marker | -9,66 | 13 | [69] |
| *TIMGD1* | T Cell Immune Regulator 1 | -10,96 | 6 | [69] |
| *UGT1A10* | UDP Glucuronosyltransferase Family 1 Member A10 | -5,31 | 6 | [69] |
| *UGT1A8* | UDP Glucuronosyltransferase Family 1 Member A8 | -5,96 | 23 | [69] |
| *UGT1A8* | UDP Glucuronosyltransferase Family 1 Member A8 | -13,65 | 31 | [70] |

% methyl: indicates % difference of DNA methylation normal to UC

#c: number of methylated cytosines
