# Supplementary material for: Toward Precision Medicine: Molecular Biomarkers of Response to Tofacitinib in Inflammatory Bowel Disease
Source: Genes (Basel). 2025 Jul 29;16(8):908. doi: 10.3390/genes16080908 (PMC12385345; doi:10.3390/genes16080908)
Supplement: Supplementary file 1 [file genes-16-00908-s001.zip › Table_S2.pdf]

Table S2: TOP hypomethylated genes in UC as reported by Taman et al. [69,70]

Gene symbol	Gene name	% methyl	#c	Ref
AQ9	Aquaporin 9	6,39	17	[69]
ADGRE3	Adhesion G Protein-Coupled Receptor E3	9,92	82	[70]
ANGPTL2	Angiopoietin-Like 2	12,71	114	[70]
CARD6	Caspase Recruitment Domain Family Member 6	25,09	20	[70]
CD300A	CD300a Molecule	9,54	94	[70]
CD300E	CD300e Molecule	12,09	30	[70]
CD48	CD48 Molecule	15,17	36	[70]
CD53	CD53 Molecule	12,03	37	[70]
CD86	CD86 Molecule	14,53	27	[70]
CD93	CD93 Molecule	14,18	102	[70]
CFP	CFP	13,67	96	[69]
CLMP	CXADR-Like Membrane Protein	11,13	213	[70]
C2CD4A	C2 Calcium Dependent Domain Containing 4A	2,43	64	[69]
C3AR1	Complement C3a Receptor 1	12,06	27	[70]
CASS6	Caspase Recruitment Domain Family Member 6	14,72	72	[70]
CD300E	CD300e Molecule	13,58	11	[69]
CH3L2	Chitinase 3 Like 2	11,96	38	[69]
CHRDL2	Chordin Like 2	10,62	65	[69]
CSF2RB	Colony Stimulating Factor 2 Receptor Beta	7,06	59	[70]
CST7	Cystatin F	11,59	39	[70]
CTSK	Cathepsin K	8,98	41	[70]
CSFR3	Colony Stimulating Factor 3 Receptor	13,10	89	[70]
CXCL5	C-X-C Motif Chemokine Ligand 5	18,85	30	[69]
CXCL6	C-X-C Motif Chemokine Ligand 6	11,03	10	[69]
CXCR1	C-X-C Motif Chemokine Receptor 1	6,08	6	[69]
CXCR2	C-X-C Motif Chemokine Receptor 2	11,60	20	[69]
CXCR2	C-X-C Motif Chemokine Receptor 2	8,67	48	[70]
DEFA6	Defensin Alpha 6	10,44	13	[69]
DMBT1	Deleted in Malignant Brain Tumors 1	11,24	45	[69]
DNAH17	Dynein Axonemal Heavy Chain 17	6,79	90	[70]
DOK3	Docking Protein 3	7,22	107	[70]
FAM124B	Family With Sequence Similarity 124 Member B	19,16	71	[70]
FCN1	Ficolin 1	8,55	12	[69]
FFAR2	Free Fatty Acid Receptor 2	2,16	54	[69]
GABRP	Gamma-Aminobutyric Acid Type A Receptor Pi Subunit	7,43	16	[69]
GNAI2	G Protein Subunit Alpha I2	5,02	109	[70]
GPSM3	G-Protein Signaling Modulator 3	4,29	54	[70]
GZMB	Granzyme B	12,88	36	[69]
HCAR2	Hydroxycarboxylic Acid Receptor 2	9,48	26	[69]
HCAR3	Hydroxycarboxylic Acid Receptor 3	14,16	18	[69]
IL17A	Interleukin 17A	9,08	29	[69]
IL1B	Interleukin 1 Beta	9,15	13	[69]
IL10	Interleukin 10	8,18	27	[70]
IL1RN	Interleukin 1 Receptor Antagonist	7,36	67	[70]
IL18R1	Interleukin 18 Receptor 1	6,91	36	[70]
ITGB2	Integrin Subunit Beta 2	20,99	98	[70]
ITPRIP	Inositol 1,4,5-Trisphosphate Receptor Interacting Protein	26,95	47	[70]

LAIR1	Leukocyte Associated Immunoglobulin Like Receptor 1	9,22	116	[70]
LCN2	Lipocalin 2	10,26	32	[69]
LYPD5	LY6/PLAUR Domain Containing 5	3,93	6	[69]
LILRA1	Leukocyte Immunoglobulin Like Receptor A1	9,66	85	[70]
LILRB1	Leukocyte Immunoglobulin Like Receptor B1	8,00	87	[70]
LILRB2	Leukocyte Immunoglobulin Like Receptor B2	25,59	74	[70]
LINC00877	Long Intergenic Non-Protein Coding RNA 877	6,15	94	[70]
LST1	Leukocyte Specific Transcript 1	14,70	40	[70]
MYO1G	Myosin IG	19,29	69	[70]
NFE2	Nuclear Factor, Erythroid 2	14,94	40	[70]
NKG7	Natural Killer Cell Granule Protein 7	13,37	61	[70]
NLRC4	NLRC4	11,95	27	[70]
NLRP12	NLR Family Pyrin Domain Containing 12	18,19	67	[70]
NLRP3	NLR Family Pyrin Domain Containing 3	17,31	70	[70]
OLFM4	Olfactomedin 4	10,95	14	[69]
OSM	Oncostatin M	22,21	34	[69]
PI3	Peptidase Inhibitor 3	13,97	10	[69]
P2RY13	Purinergic Receptor P2Y13	8,88	15	[70]
PLEKHO1	Pleckstrin Homology Domain Containing O1	21,61	50	[70]
PPP1R18	Protein Phosphatase 1 Regulatory Subunit 18	11,27	147	[70]
PTPRC	Leucocyte Common Antigen	13,83	18	[70]
REG1B	Regenerating Family Member 1 Beta	6,63	7	[69]
REG3A	Regenerating Family Member 3 Alpha	7,27	2	[69]
ROH	Ras Homolog Family Member H	21,01	45	[70]
S100A9	S100 Calcium Binding Protein A9	8,26	40	[69]
SAA1	Serum Amyloid A1	10,55	26	[69]
SAA2	Serum Amyloid A2	1,89	8	[69]
SCARF1	Scavenger Receptor Class F Member 1	19,10	97	[70]
SLC26A4	Solute Carrier Family 26 Member 4	5,22	54	[69]
SLC6A14	Solute Carrier Family 6 Member 14	3,99	29	[69]
SELPLG	Selectin P Ligand	11,57	78	[70]
SEMA4A	Semaphorin 4A	20,45	47	[70]
SIGLEC5	Sialic Acid Binding Ig Like Lectin 5	8,02	54	[70]
SLA	Src-Like-Adaptor	20,31	35	[70]
SLAMF1	SLAM Family Member 1	15,47	44	[70]
SLAMF7	SLAM Family Member 7	9,93	21	[70]
SLAMF8	SLAM Family Member 8	11,27	46	[70]
SNX20	Sorting Nexin 20	22,87	48	[70]
SPARC	Secreted Protein Acidic And Cysteine Rich	13,47	72	[70]
SPI1	Spleen Focus Forming Virus Proviral Integration Oncogene	10,81	41	[70]
TIE1	Tyrosine Kinase With Immunoglobulin Like And EGF Like Domains 1	9,44	52	[70]
TNFSF14	TNF Superfamily Member 14	13,04	122	[70]
TNFSF8	TNF Superfamily Member 8	12,16	71	[70]
TREML2	Triggering Receptor Expressed On Myeloid Cells Like 2	7,52	48	[70]
WARS	Tryptophanyl TRNA Synthetase	3,28	31	[70]

% methyl: indicates % difference of DNA methylation normal to UC

#c: number of methylated cytosines
