# Supplementary material for: Toward Precision Medicine: Molecular Biomarkers of Response to Tofacitinib in Inflammatory Bowel Disease
Source: Genes (Basel). 2025 Jul 29;16(8):908. doi: 10.3390/genes16080908 (PMC12385345; doi:10.3390/genes16080908)
Supplement: Supplementary file 1 [file genes-16-00908-s001.zip › Table_S3.pdf]

Table S3: TOP CpGs predictors of response to TOFA reported by Joustra et al. [71]

CpG	Associated gene	Gene name	Methylation	LTS
cg20611115	*MAGI3*	Membrane Associated Guanylate Kinase Inverted 3	Hyper	NA
cg21242009			Hyper	0,89
cg11757444	*RPTOR*	Regulatory Associated Protein Of MTOR Complex 1	Hypo	NA
cg21650737	*ERICH1*	Glutamate Rich 1	Hyper	0,84
cg20758756			Hyper	0,99
cg10760651	*CLCA1*	Chloride Channel Accessory 1	Hypo	0,93
cg00598125	*HLA-DRB1* *HLA-DRB5*	HLA class II histocompatibility antigen	Hypo	0,97
cg24593918	*HLA-DQB1*	HLA class II histocompatibility antigen	Hyper	0,98
cg07237217	*PALD1*	Phosphatase Domain Containing Paladin 1	Hyper	0,97
cg26725559			Hyper	0,94
cg08944170	*OR2L13*	Olfactory Receptor Family 2 subfamily L Member 13	Hypo	0,99
cg20507276	*OR2L13*	Olfactory Receptor Family 2 subfamily L Member 13	Hypo	0,99
cg26207766			Hyper	0,93
cg03748376	*OR2L13*	Olfactory Receptor Family 2 subfamily L Member 13	Hypo	0,99
cg03418136	*RP11-526l2.5*	Pseudogene	Hypo	0,98
cg18990612			Hyper	0,94
cg10788837			Hyper	0,97
cg05200811	*SPATC1L*	Spermatogenesis And Centriole Associated 1 Like	Hypo	0,98
cg17422692	*MRPL28*	Mitochondrial Ribosomal Protein L28	Hypo	0,93
cg17152205			Hyper	0,99
cg24630035			Hypo	0,92
cg11862180			Hyper	0,96
cg00299286	*BTNL9*	Butyrophilin-like 9	Hyper	0,97
cg22746029	*LMLN2*	Leishmanolysin like peptidase 2	Hyper	0,96
cg02270332			Hypo	0,97
cg22180157	*KIAA1671*	Uncharacterized protein KIAA1671	Hypo	NA
cg24760581	*HLA-DRB5*	HLA class II histocompatibility antigen	Hypo	NA
cg18797653	*VTRNA2-1*	Vault RNA 2-1	Hyper	0,97
cg16474696	*MRI1*	Methylthioribose-1-Phosphate Isomerase 1	Hyper	0,99
cg05194426	*CYP2E1*	Cytochrome P450 2E1	Hypo	0,97
cg10075506	*MyT1L*	myelin transcription factor 1 like	Hypo	0,98
cg22992730			Hyper	0,95
cg07469075	*PAMR1*	Peptidase Domain Containing Associated With Muscle Regeneration 1	Hypo	0,95
cg15146462			Hypo	0,9
cg10518850	*LRPAP1*	LDL Receptor Related Protein Associated Protein 1	Hyper	0,91
cg22000984	*IRGM*	Immunity-related GTPase family M protein	Hypo	0,96
cg24543140			Hypo	NA
cg25279747			Hyper	0,97
cg20246743			Hyper	0,98
cg01077499	*AX746535*	Unknown function	Hypo	NA
cg23540632	*DOK6*	Docking Protein 6	Hyper	0,97
cg24717262	*SGCD*	Sarcoglycan Delta	Hypo	NA
cg00347798	*CLEC3B*	C-Type Lectin Domain Family 3 Member B	Hyper	0,87

cg14780466	*GDF7*	Growth Differentiation Factor 7	Hypo	0,97
cg26095158	*LYPLAL1-DT*	LYPLAL1 Divergent Transcript	Hypo	0,98
cg17046825	*CRYL1*	Crystallin Lambda 1	Hypo	NA
cg01283332			Hypo	0,94
cg18822299	*DDX10*	DEAD-Box Helicase 10	Hyper	0,97
cg26805839	*SLC1A1*	Solute Carrier Family 1 Member 1	Hyper	0,98
cg07102913	*PCDHB13*	Protocadherin beta 13	Hypo	0,55
cg00791851			Hypo	0,94
cg17107388	*NDRG4*	NDRG Family Member 4	Hyper	0,95
cg08899523	*FGFR2*	Fibroblast growth factor receptor 2	Hyper	0,98

Hyper: Hypermethylation

Hypo: Hypomethylation

LTS: Long term stability
